# Corrected QT interval prolongation after ganglionated plexus ablation: myth or reality?—Authors’ reply

**DOI:** 10.1093/europace/euab148

**Published:** 2021-06-23

**Authors:** Ben J M Hermans, Matthias D Zink, Frank van Rosmalen, Harry J G M Crijns, Kevin Vernooy, Pieter Postema, Laurent Pison, Ulrich Schotten, Tammo Delhaas

**Affiliations:** 1 Department of Biomedical Engineering, Cardiovascular Research Institute Maastricht (CARIM), Maastricht University, PO Box 616, 6200 MD Maastricht, The Netherlands; 2 Department of Physiology, Cardiovascular Research Institute Maastricht (CARIM), Maastricht University, Maastricht, The Netherlands; 3 Department of Cardiology, Angiology and Intensive Care Medicine, University Hospital RWTH Aachen, Aachen, Germany; 4 Department of Cardiology, Cardiovascular Research Institute Maastricht (CARIM), Maastricht University Medical Center, The Netherlands; 5 Department of Cardiology, Heart Center, Amsterdam UMC, University of Amsterdam, Amsterdam, The Netherlands; 6 Department of Cardiology, Ziekenhuis Oost, Limburg, Genk, Belgium

We thank Prof. Aksu for this valuable and well-balanced discussion on the possible effect of ablation of atrial ganglionated plexuses (GPs) on the QT interval.^1^ Their comments are in line with our statement that from our study^2^ we neither can conclude that pulmonary vein isolation (PVI) does not modulate GP nor that GP modulation leads to changes in QTc. We can, however, conclude that, on average, routine PVI does not induce changes in QTc.

**Conflict of interest:** none declared.
